# Inhibitory Effects of Probiotic *Lactobacillus* on the Growth of Human Colonic Carcinoma Cell Line HT-29

**DOI:** 10.3390/molecules22010107

**Published:** 2017-01-10

**Authors:** Zhung-Yuan Chen, You-Miin Hsieh, Chun-Chih Huang, Cheng-Chih Tsai

**Affiliations:** 1 Department of Food and Nutrition, Providence University, Taichung City 43301, Taiwan; g1010046@pu.edu.tw (Z.-Y.C.); ymhsieh@pu.edu.tw (Y.-M.H.); 2 New Bellus Enterprises Co., Ltd. No. 48, Industrial Rd., Erh Chen Vil., Kuan Tien Dist., Tainan City 72042, Taiwan; john@newbellus.com.tw; 3 Department of Food Science and Technology, Hungkuang University, Taichung City 43302, Taiwan

**Keywords:** *Lactobacillus*, HT-29 cells, interleukin-8, Bax/Bcl-2 ratio, lactate dehydrogenase

## Abstract

This study was conducted to investigate the inhibitory effect of *Lactobacillus* cells and supernatants on the growth of the human colon cancer cell line HT-29. Our study results indicated that the PM153 strain exhibits the best adhesion ability and the highest survival in the gastrointestinal tract simulation experiment. Furthermore, after an 8-h co-culture of PM153 and HT-29 cells, the PM153 strain can induce the secretion of nitric oxide from the HT-29 cells. In addition, after the co-culture of the BCRC17010 strain (10^9^ cfu/mL) and HT-29 cells, the Bax/Bcl-2 ratio in the HT-29 cells was 1.19, which showed a significant difference from the other control and LAB groups (*p* < 0.05), which therefore led to the inference that the BCRC17010 strain exerts a pro-apoptotic effect on the HT-29 cells. Upon co-culture with HT-29 cells for 4, 8 and 12 h, the BCRC14625 strain (10^9^ cfu/mL) demonstrated a significant increase in lactate dehydrogenase (LDH) activity (*p* < 0.05), causing harm to the HT-29 cell membrane; further, after an 8-h co-culture with the HT-29 cells, it induced the secretion of nitric oxide (NO) from the HT-29 cells. Some lactic acid bacteria (LAB) strains have ability to inhibit the growth of the colorectal cancer cell line HT-29 Bax/Bcl-2 pathway or NO production. In summary, we demonstrated that the BCRC17010 strain, good abilities of adhesion and increased LDH release, was the best probiotic potential for inhibition of HT-29 growth amongst the seven LAB strains tested in vitro.

## 1. Introduction

Colorectal cancer is the second leading cause of death due to cancer in the United States [1]. The incidence rate of colorectal cancer increases with age. Excessive alcohol consumption may lead to an increase in the colorectal cancer incidence rate by 60%; insufficient fibre intake and smoking may increase the colorectal cancer incidence rate by 20%. Previous research has found that the excessive intake of high-fat diets can also lead to colorectal cancer [2]. At least 15%–20% of cancers are attributed to infection or inflammation, including malignant tumours such as colorectal cancer and inflammatory bowel disease (including ulcerative colitis and Crohn’s disease). Research has suggested that patients with the above infections are more likely to develop colorectal cancer [3].

Probiotics are live microorganisms and can exert health benefits to their hosts when ingested in sufficient quantities [4]. The fundamental criteria for probiotic selection include safety (must be a generally recognised as a safe strain), acid and bile salt tolerance, ability to adhere to the intestinal tract and colonise and exhibit health benefits to the host [5,6]. The most common effects of lactobacilli are to reduce the activity of tumour-promoting enzymes, increase host immunity, produce metabolites that benefit the host and resist pathogens.

In vitro human intestinal cell models have been used to study the function of specific intestinal cells in humans, with Caco-2 and HT-29 the most widely used human intestinal cell lines [7]. Caco-2 cell lines and clones display a spontaneous differentiation-dependent enterocyte-like phenotype of the small intestine [8]. The parental colon cancer HT-29 cell line is composed mainly of undifferentiated cells, with a small minority of differentiated cells (~3% to 5% of total cells) [8]. *Lactobacillus helveticus*, *Bifidobacterium* or a mix of *Streptococcus thermophilus* and *L. bulgaricus* significantly reduced the growth rate of HT-29 cells, resulting in a 10%–50% decrease in the total cell number. The most effective strains in lowering the HT-29 growth rate were *L. helveticus* and *Bifidobacterium* [9]. In *Lactobacillus* or *Bifidobacterium*-fermented soy milk, soy isoflavones such as genistein and daidzein and cell walls and extracellular components of the two probiotics engaged in the interaction with tumours and inhibited tumour growth [10,11].

The inhibitory mechanisms of *Lactobacillus* against colorectal cancer cells include reducing tumour-promoting enzymatic activity, binding to mutagens, increasing short-chain fatty acids, lowering pH and enhancing immunity [12,13,14,15]. This study aimed to investigate the probiotic characteristics and their ability to inhibit the growth of the colorectal cancer cell line HT-29 with detection of Bax/Bcl-2, LDH and NO.

## 2. Results

### 2.1. Analysis of Probiotic Characteristics of Lactobacillus

**Figure 1:**
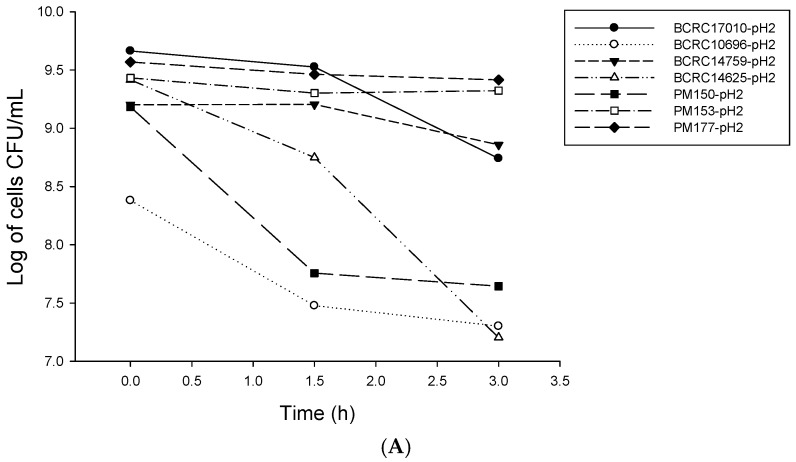

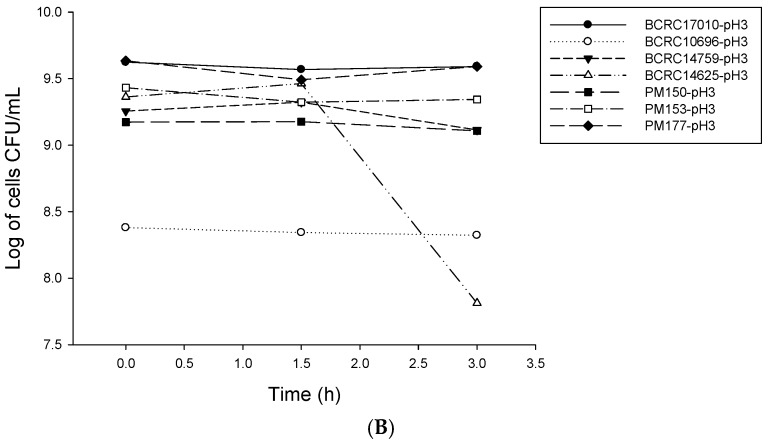
Survival of lactic acid bacteria in simulated gastric juice (**A**) pH 2.0 (**B**) pH 3.0.

The simulation experiment of human gastrointestinal tract tolerance of *Lactobacillus* was used to assess the tolerance of *Lactobacillus* to gastrointestinal tract conditions. For all strains cultured in simulated gastric juice at pH 2 for 0, 1.5 and 3 h (Figure 1A), following a 3-h culture, the numbers of PM177, PM153, BCRC17010 and BCRC14759 were maintained above 10^8^ cfu/mL, indicating fairly good acid tolerance. In addition, for all strains cultured in simulated gastric juice at pH 3 for 0, 1.5 and 3 h, except for BCRC14625 that exhibited a nearly 2-log reduction in the number of viable bacteria, the numbers of all other six *Lactobacillus* strains were maintained within 10^9^ cfu/mL (Figure 1B).

By microscopic observations of the number (mean ± SD) of *Lactobacillus* cells attached to a single HT-29 cell, the adhesion abilities of seven *Lactobacillus* strains to the HT-29 cells were as follows: PM153 (15.6 ± 5.02 bacterial cells/cell), BCRC17010 (9.2 ± 4.73 bacterial cells/cell), PM177 (7.6 ± 2.76 bacterial cells/cell), BCRC14625 (5.2 ± 3.36 bacteria cells/cell), PM150 (5.2 ± 3.12 bacterial cells/cell) and BCRC10696 (4.2 ± 3.36 bacterial cells/cell); BCRC14759 was unable to adhere to the HT-29 cells.

### 2.2. Lactobacillus Supernatants Inhibit the Viability of HT-29 Cells

In our experiments, the MTT assay was used to determine the inhibitory effect of *Lactobacillus* supernatants on HT-29 cells. Table 1 shows the pH values and l-lactic acid contents of the supernatants of the seven *Lactobacillus* strains. The pH values were ranged between 3.73 and 4.25. Strains BCRC17010, PM153 and PM177 showed the highest l-lactic acid levels. Table 2 shows the inhibitory effects of the MRS medium under difference pH values (pH 4.5, 5.5, 6.5, 7.5) and l-lactic acid levels (10, 50, 100, 150, 200 mM) on the growth of HT-29 cell lines using MTT assay. The inhibition ratio (%) increased when decreased the pH value or increased l-lactic acid levels. The supernatants from the seven strains of lactobacilli were adjusted to pH 7 and were then added in various concentrations of 200, 300, 400, 500, 600 and 700 μL/mL onto the HT-29 cells, which was then followed by a 24-h culture. Table 3 shows that the IC 50 values for the HT-29 cells treated with supernatants from the seven *Lactobacillus* strains are 479.2 μL/mL (BCRC17010), 609.8 μL/mL (BCRC10696), 370.7 μL/mL (BCRC14625), 467.9 μL/mL (BCRC14759), 667.5 μL/mL (PM150), 299.3 μL/mL (PM153) and 134.9 μL/mL (PM177). The above results reveal that PM177 exerts the best inhibitory effect, whereas PM150 exerts the worst.

### 2.3. Damage to HT-29 Cells Caused by Lactobacillus Supernatants and Cells

**Figure 2:**
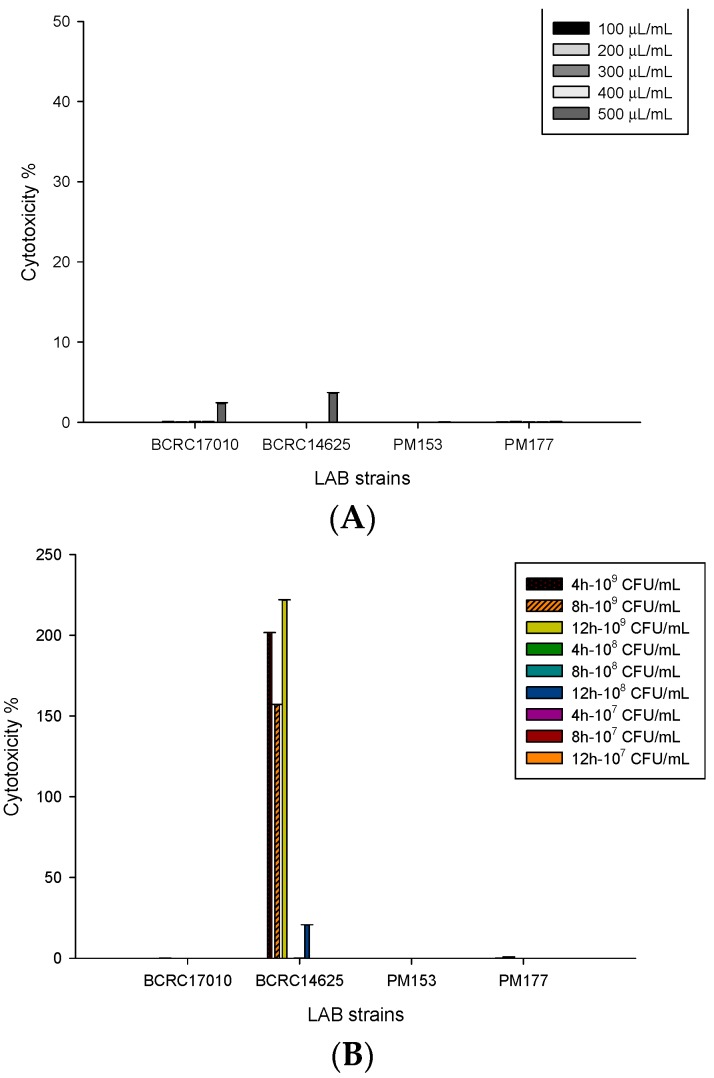
(**A**) Measurement of cytotoxicity on HT-29 cell line by lactic acid bacteria supernatants after 24 h incubation; (**B**) Measurement of cytotoxicity (%) on HT-29 cell line by lactic acid bacteria strains after 4, 8, 12 h incubation. cytotoxicity (%) = [(Experimental OD_490_ − Spontaneous negative control OD_490_)/Maximum LDH release positive control OD_490_] × 100.

The amount of LDH released is used to determine the degree of damage in HT-29 cell membranes caused by *Lactobacillus* supernatants in different concentrations and by *Lactobacillus* cells in different numbers. The results showed that the supernatants (500 μL/mL) of BCRC17010 and BCRC14625, compared to those in other concentrations, induced a significant increase in LDH concentration, indicating that supernatants of high concentration will damage the HT-29 cell membrane causing LDH release (Figure 2A). For *Lactobacillus* cells, the LDH level was significantly increased (*p* < 0.05) upon co-culture of BCRC14625 (10^9^ cfu/mL) with HT-29 cells for 4, 8 and 12 h. Similarly, the LDH level was also elevated (*p* < 0.05) upon co-culture of 10^8^ cfu/mL of BCRC14625 and HT-29 cells for 12 h. Thus, it can be concluded that BCRC14625 damages the HT-29 cell membrane, leading to the release of LDH, and that lower numbers of bacteria would take longer to cause harm in the cell membrane (Figure 2B).

### 2.4. Apoptosis-Associated Protein Analysis in HT-29 Cells Cultured with Lactobacillus Supernatants and Lactobacillus Cells

**Figure 3:**
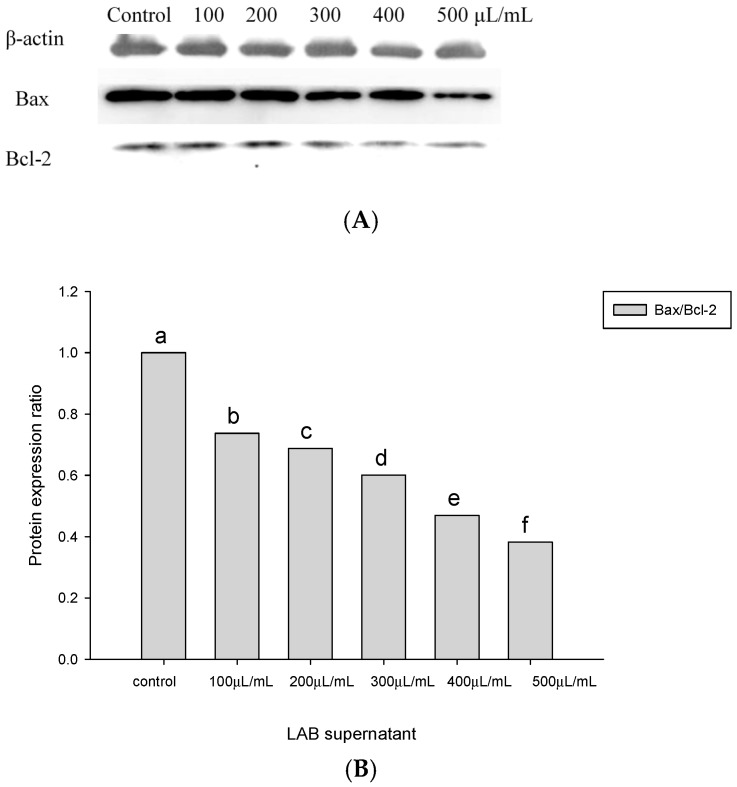
Effect of BCRC14625 supernatant on the level of Bax/Bcl-2 in the HT-29 cell line. The control means untreated cells. (**A**) Protein products of Bax and Bcl-2 extracted from the HT-29 cell line; (**B**) Bars represent the protein expression ratio of Bax/Bcl-2 in the HT-29 cell line.

**Figure 4:**
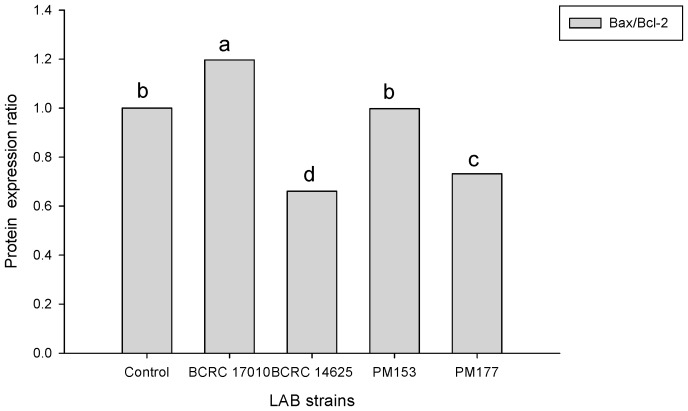
Four lactic acid bacteria (10^9^ cfu/mL) in HT-29 for 8 h after on the level of Bax/Bcl-2 in HT-29 cell line. The control means untreated cells. ^a–d^ Values with different symbols are significantly different at the level of *p* < 0.05 by One-way ANOVA.

The ratios of Bax/Bcl-2 protein expression in the HT-29 cells incubated with BCRC14625 were less than 1 (Figure 3). To assess the effect of *Lactobacillus* cells on HT-29 cell apoptosis, the ratio of Bax and Bcl-2 protein expression was determined after incubating the HT-29 cells with 10^9^ cfu/mL of *Lactobacillus* cells for 8 h. The Bax/Bcl-2 ratio in response to BCRC17010 was 1.19, which was significantly different from that in response to other strains (*p* < 0.05), demonstrating that the BCRC17010 cells showed a pro-apoptotic effect in the HT-29 cells (Figure 4).

### 2.5. Capabilities of Lactobacillus Cells and Their Supernatants to Induce NO Production in HT-29 Cells

**Figure 5:**
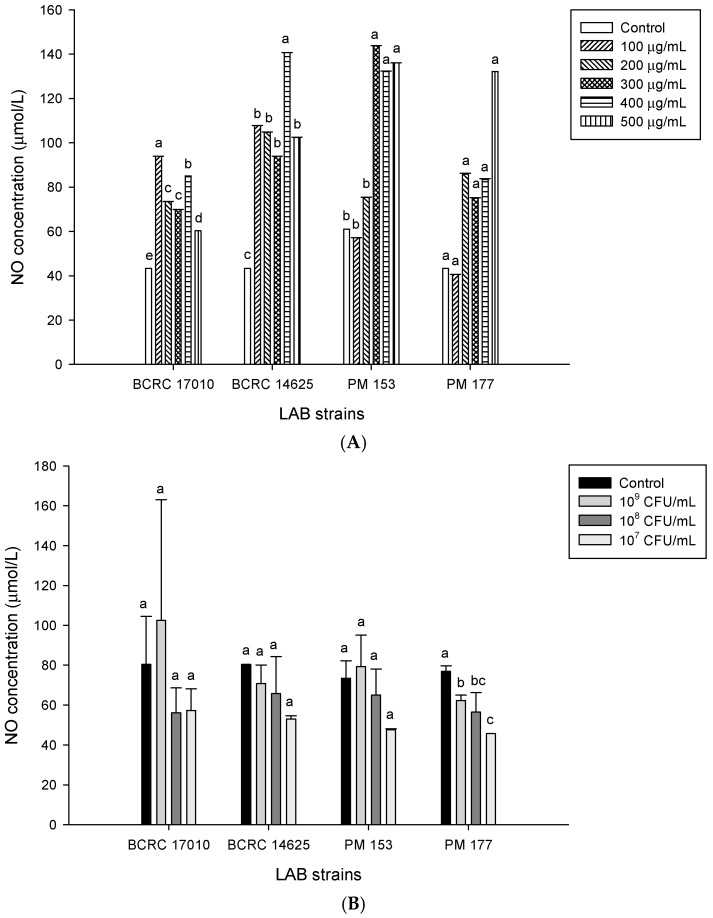

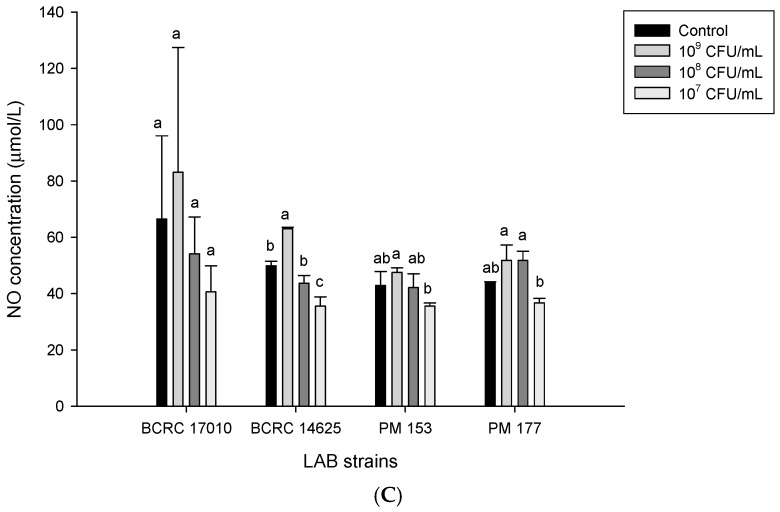
NO production was used by (**A**) lactic acid bacteria supernatant on HT-29 cell line after 24 h or four lactic acid bacteria strains on HT-29 cell line after (**B**) 4 h (**C**) 8 h incubation. The control means untreated cells. Data are expressed as means ± SD, ^a–e^ Values with different symbols are significantly different in the same LAB strain group at the level of *p* < 0.05 by One-way ANOVA.

Our study showed that after treating the HT-29 cells separately with supernatants from four different lactobacilli strains for 24 h, NO concentrations in the *Lactobacillus*-induced and control groups were significantly increased (Figure 5A). When comparing the results of cell viability and NO production in the *Lactobacillus* supernatant-treated HT-29 cells, we discovered that the higher the supernatant concentration was, the lower was the cell viability, which may be attributed to the fact that the supernatant caused HT-29 cell death via the production of NO. In terms of *Lactobacillus* cells, after a 4-h treatment of the HT-29 cells with the *Lactobacillus* strains BCRC17010, BCRC14625, PM153 and PM177, the corresponding NO concentrations were not significantly different from that in the control group (*p* > 0.05) (Figure 5B). The concentration of NO was also analysed after treating the HT-29 cells with individual *Lactobacillus* strains, showing that the NO concentration induced by BCRC14625 (10^9^ cfu/mL) was significantly higher than that in the control group (*p* < 0.05) (Figure 5C).

## 3. Discussion

Conway et al. [16] found that the viability of *Lactobacillus* is lower in PBS than in gastric juice because certain components in gastric juice may confer protective effects on bacterial cells. Research has suggested that the acid tolerance of *Lactobacillus* is attributed to the presence of a constant gradient between extracellular and cytoplasmic pH [17]. Under acidic conditions in the absence of sugars, lactobacilli produce less ATP, revealing that sugar is an important factor affecting *Lactobacillus* viability [18]. In the simulated intestinal fluid experiment to examine bile salt tolerance, the reason for the decrease in bacterial numbers may be damage to bacterial lipid membranes by bile salts, which thus influences the integrity of bacterial cell membranes [19]. Additionally, bile salts can affect the expression of genes responsible for maintaining the cytoplasm and cell wall in lactobacilli [20]. Huang et al. [21] indicated that after culturing *L. acidophilus* BCRC10695, *L. paracasei* BCRC14023 and *B. bifidum* BCRC14615 in 0.1% peptone water with added 0.1, 0.2 and 0.3% (*w*/*v*) bile salts for 1.5 and 3 h, respectively, the viability of *B. bifidum* BCRC14615 after the 3-h culture with 0.2 and 0.3% (*w*/*v*) bile salts approached zero and the viability of the other two strains also decreased with elevated bile salt concentrations.

The adhesion ability of probiotic lactobacilli has been considered significant in the colonisation of bacteria within the gastrointestinal tract and in the beneficial benefits of bacteria on the hosts. Tuo et al. [22] demonstrated that *L. rhamnosus* IN1L had the highest adhesive capability (251 ± 14 bacterial cells/100 cells) to the HT-29 cells; *L. rhamnosus* J5L had the second highest (32.3 ± 2.5 bacterial cells/100 cell) and *L. rhamnosus* SB5L and *L. rhamnosus* SB31L had the worst (2.5 ± 1.1, 2.6 ± 0.4 bacterial cells/100 cells) adhesive capabilities. In our study, except for BCRC14759 that had no adhesion capability, the other six strains of lactobacilli exhibited better adhesion capabilities to the HT-29 cells than those used in the above literature.

Lan et al. suggested that some probiotic activities result from the organic acids level and pH value of probiotics cultures [23]. However, Motevaseli et al. demonstrated that the lactic acid level was a more important part of the lactobacilli inhibitory effect than pH alone [24]. In the present study, HT-29 cells line growth inhibition was by the pH value, lactic acid level as well as culture supernatants. Sadeghi-Aliabadi et al. [25] determined the influence of *L. plantarum* A7 and *L. rhamnosus* GG supernatants in three concentrations (2.5, 5 and 10 mg/mL) on HT-29 cell viability. Their results have indicated that the viabilities of HT-29 cells cultured with the *L. plantarum* A7 supernatants were 46% ± 2.6%, 15% ± 7%, and 0%, respectively. Under the same condition, the viabilities of HT-29 cells cultured with *L. rhamnosus* GG supernatants were 54% ± 14%, 19% ± 2.9%, and 0.4% ± 0.75%, respectively. Furthermore, a significant difference in HT-29 cell viability was found between the group with added 10 mg/mL *Lactobacillus* supernatants and the control group, indicating that in the comparison of the original supernatant with the supernatant adjusted to pH 7, the lower pH has a greater impact on the growth inhibition of the HT-29 cells.

Wang et al. [26] suggested that lactobacilli isolated from cheese induce cell apoptosis. Sun et al. [27] showed that in the intestinal epithelium, the ratio of Bax and Bcl-2 protein expression can serve as an index of the mitochondria-mediated apoptotic pathway, with a ratio of more than 1 indicating that the tested molecules exert pro-apoptotic effects. Khoury et al. [28] reported that kefir significantly upregulates the ratio of Bax and Bcl-2 in HT-29 and Caco-2 cells, indicating that kefir promotes cell apoptosis in the HT-29 and Caco-2 lines. Iyer et al. [29] revealed that *L. reuteri* may participate in the *Lactobacillus*-induced extrinsic pathway of apoptosis to prevent the occurrence of colorectal cancer. However, although BCRC14625, a strain of *L. reuteri* used in our study, and the strain in the aforementioned literature both belong to the same species, BCRC14625 does not exhibit the ability to induce cell apoptosis.

*Lactobacillus* can produce NO by utilising nitrate and nitrite, thus exhibiting immunoregulatory and antibacterial effects [30]. NO has a dual function: damaging DNA to cause cell death and inducing cell apoptosis [31]. Literature has also indicated that NO inhibits Bcl-2 [32]. Based on our findings, following the 24-h treatment of the HT-29 cells with the BCRC14625 supernatants at various concentrations, the corresponding amounts of Bcl-2 were significantly lower than that in the control group (Figure 3A), providing evidence that NO can inhibit the anti-apoptotic protein Bcl-2.

## 4. Materials and Methods

### 4.1. Bacteria Strains, Culture Medium and Growth Conditions

A total of 7 strains of *Lactobacillus*, including *L. johnsonii* BCRC17010, *L. delbrueckii* subsp. *bulgarius* BCRC10696, *L. salivarius* BCRC14759, *L. reuteri* BCRC14625, *L. brevis* PM150, *L. plantarum* PM153 and *L. brevis* PM177, were obtained from isolated strains of *Lactobacillus* found in fermented plant products (PM150, PM153 and PM177) or from the Bioresource Collection and Research Center (BCRC), Hsin-Chu, Taiwan. To prepare *Lactobacillus* strains for use in this study, *Lactobacillus* stored at −80 °C was inoculated into *Lactobacilli* MRS broth (Difco) containing 0.05% l-cysteine and activated two times prior to incubation for 20 h at 37 °C before use. The l-lactic acid was detected by National Standards of the Republic of China (CNS) 12635 N6224 method.

### 4.2. LAB Resistance to Simulated Gastrointestinal Conditions

One milliliter of culture containing approximately 10^9^ cfu/mL of LAB was centrifuged (1000× *g*, 10 min) and added into 10 mL phosphate-buffered saline (PBS) with 0.3% Pepsin (Sigma-Aldrich, St. Louis, MO, USA). The pH was adjusted to 2.0, 3.0 and 7.0 using 0.1 N HCl, and the solution was incubated at 37 °C for 0, 1.5 and 3 h. After incubation, viable bacterial counts were determined by serially diluting the culture in PBS (pH 7.2) and plating on MRS agar. Plates were incubated anaerobically at 37 °C for 48 h.

### 4.3. The Epithelial Cell Line Culture and Adhesion Assay

The HT-29 cell line was obtained from BCRC, Hsin-Chu, Taiwan. HT-29 cells were grown in Eagle’s minimal essential medium (EMEM) (GIBCO) supplemented with 10% (*v*/*v*) fetal bovine serum, 1% nonessential amino acid (NEAA), and 50 unit·mL^−1^ Penicillin-Streptomycin (GIBCO). The HT-29 cell line was cultured in 75 cm^2^ plastic tissue culture flasks (GIBCO). The cells were washed twice with PBS and then transferred (4 × 10^5^ cells/mL) with 0.05% trypsin into a 24-well multi-dish containing fresh tissue culture medium without Penicillin-Streptomycin. The mixtures were kept at 37 °C in 5% CO_2_/95% air atmosphere until cell lines formed a monolayer in each well. Prior to the adhesion test all bacterial strains were washed twice with PBS and centrifuged for 10 min at 8000 × *g*. One milliliter of broth inoculated with *Lactobacillus* was centrifuged for 10 min, washed with 1 × PBS buffer and re-dissolved in cell culture medium without antibiotics. Then, 100 μL of the broth was added to a 24-well plate and incubated in a 37 °C CO_2_ incubator for 2 h. After incubation cells were washed twice with PBS, fixed with 10% formalin for 30 min, washed four times with PBS and then stained with crystal violet for 5 min. The numbers of LAB cells adhered to the cultured cell lines were counted according to the method of [33].

### 4.4. Analysis of Cell Viability

This experiment focused on analysing the ability of *Lactobacillus* to inhibit colorectal cancer cells. 3-(4,5-Dimethylthiazol-2-yl)-2,5-diphenyltetrazolium bromide (MTT), a colourless, transparent tetrazolium salt, is reduced to yield a purple formazan crystal by mitochondrial dehydrogenase in living cells. In total, 500 μL (10^4^ cells/mL) of cells was seeded into a 24-well plate, and the cells were subjected to overnight culture at 37 °C in a CO_2_ incubator, which made the cells attach, divide and grow in the 24 wells. The cells were gently washed twice with 1× PBS, and after discarding PBS, 1 mL of mixed solutions of *Lactobacillus* culture supernatants and cell culture media were added to the respective wells. The results were subsequently analysed after 24 h. For the analysis, first, the solution was aspirated from the 24-well plate, and the cells were gently washed with 1× PBS twice, followed by the removal of PBS by suction. Second, 300 μL of MTT solution was added to the cells. After a 1-h culture at 37 °C in a CO_2_ incubator, the supernatants were removed and 200 μL of dimethyl sulfoxide was added to the wells, which was followed by continuous shaking for 10 min to solubilise the purple formazan crystals. An ELISA reader (Model 680, BIO-RAD, Hercules, CA, USA) was used to read the absorbance at 570 nm; next, inhibitory rates were calculated to determine IC_50_ values. The formula to calculate the inhibitory rate is as follows: Inhibition ratio (%) = [(OD_control_ − OD_treated_)/(OD_control_)] × 100%; OD, optical density.

### 4.5. Quantitative Measurements of Bax and Bcl-2 Proteins Involved in the Apoptotic Pathway of Colorectal Cancer Cells

Apoptosis-associated protein expression in the HT-29 cells was determined by western blotting. The HT-29 cells were added to a 10-well plate and cultured overnight at 37 °C in the CO_2_ incubator to make the cells attach, divide and grow in the wells. The solutions of *Lactobacillus* supernatants in various concentrations mixed with the cell culture media were added to the wells, and the cells were cultured for 24 h at 37 °C in the CO_2_ incubator, which was followed by the addition of *Lactobacillus* cells (10^9^ cfu/mL) mixed with the cell culture media. After a 6-h culture in the CO_2_ incubator at 37 °C, the cells were collected and then lysed in radioimmunoprecipitation assay buffer for 30 min on ice. Next, the cells were centrifuged at 12,000 rpm for 5 min at 4 °C. The resulting supernatants (extracts of cellular proteins) were subjected to quantitative protein analysis using the Invitrogen Qubit^®^ fluorometer (Life Technologies, Waltham, MA, USA).

Cellular protein extracts were mixed with 5× loading dye, heated at 95 °C for 5 min and loaded in each well for SDS-PAGE. Following electrophoresis, proteins on the gel were transferred to a PVDF membrane for an hour at 37 °C with the addition of 5% skim milk as the blocking buffer. Subsequently, the membrane was washed in TBST [20 mM Tris–HCl, pH 8 and 137 mM NaCl containing 0.1% (*v*/*v*) Tween-20] three times and was incubated with the primary antibody (monoclonal antibodies of Bax and Bcl-2) overnight at 4 °C. After three washes with TBST, the secondary antibody (HRP-conjugated goat anti-mouse IgG) was added and incubated with the membrane for 1 h, which was followed by TBST washes. The resulting gel images were captured using a luminometer, and bands were observed [34].

### 4.6. Analysis of Lactate Dehydrogenase (LDH)

LDH stably exists in the cytoplasm and is released from cells with a damaged membrane; therefore, LDH activity in the cell culture medium is positively correlated with the number of necrotic cells. The CytoScan^TM^-LDH cytotoxicity assay kit (G-Biosciences, St. Louis, MO, USA) was used to measure LDH release: the HT-29 cells (10^4^ cells/100 μL) were seeded in 96 wells and cultured overnight to ensure that the cells attached and grew in the wells. After the removal of the old culture medium, the cells were washed twice with PBS (including 1% BSA) and then cultured with a series of 100 μL mixed solutions of *Lactobacillus* supernatants in different concentrations and cell culture media for 24 h at 37 °C in the CO_2_ incubator. Alternatively, the HT-29 cells were cultured with mixed solutions of 100 μL of *Lactobacillus* cells (10^9^, 10^8^ and 10^7^ cfu/mL), and following 4-, 8- and 12-h cultures at 37 °C in the CO_2_ incubator, the cells were centrifuged at 1100 rpm for 5 min. Next, 50 μL of supernatants were aspirated and transferred to a new 96-well plate. In addition, unprocessed cells in one of the 96 wells were used as the control group and cultured for 45 min at 37 °C in the CO_2_ incubator after the addition of 10 μL of 10× lysis buffer. Later, 50 μL of substrate mix was added to each well in the dark, left to stand for 20 min at 37 °C, followed by the addition of 50 μL of stop solution. The resulting absorbance in each well was measured at 490 nm.

### 4.7. Analysis of Nitric Oxide (NO) Concentration

The HT-29 cells were seeded in a 24-well plate and cultured overnight at 37 °C in the CO_2_ incubator to make the cells attach, divide and grow in the wells. The supernatants were collected, either after a 24-h co-culture at 37 °C in the CO_2_ incubator with the addition of a series of mixed solutions of *Lactobacillus* supernatants in various concentrations and cell culture media or after 4- and 8-h co-cultures at 37 °C in the CO_2_ incubator with the addition of mixed solutions of *Lactobacillus* cells (10^9^, 10^8^ and 10^7^ cfu/mL) and cell culture media.

Parameter^TM^ Total Nitric Oxide and Nitrate/Nitrite Assay (R&D Systems, Minneapolis, MN, USA) was used to determine the total amount of NO. First, the collected supernatants were diluted with a 1× reaction diluent. Next, in 96 wells, 50 μL of the reaction diluent (1×), 50 μL of the standard and sample, 25 μL of the NADH reagent, and 25 μL of nitrate reductase were added to each well and left to stand for 30 min at 37 °C. Then, 50 μL of Griess reagent Ι and 50 μL of Griess reagent ΙΙ were added to the wells, and the wells were left to stand for 10 min at room temperature. Lastly, the ELISA reader was used to measure the absorbance at 540 nm and 690 nm.

### 4.8. Statistical Analysis

Statistical analysis of the study data was performed using the SAS 9.4 statistical software (SAS Institute Inc., Cary, NC, USA). One-way analysis of variance (ANOVA) or independent sample *t*-test was used to determine the statistical significance; *p* < 0.05 indicates significant difference.

## 5. Conclusions

In conclusion, the BCRC14625 strain causes harm to the HT-29 cell membrane according to significant increase in lactate dehydrogenase (LDH) activity. LAB is considered to inhibit the growth of colonic carcinoma cells. Our findings are that the ratio of apoptotic proteins Bax: Bcl-2 for BCRC17010 significantly differed from other strains, indicating that BCRC17010 may lead to apoptosis via the apoptotic mitochondrial pathway. BCRC17010 also had good adhesion ability and significantly increased LDH release. We suggested that the strain BCRC17010 shows the best probiotic potential for inhibition of HT-29 growth amongst the seven LAB strains tested.

## Figures and Tables

**Table 1:** The pH values and l-lactic acid contents of *Lactobacillus* supernatants.

LAB Strains	pH of Supernatant	l-Lactic Acid Content (mM)
PM150	3.85 ± 0.19 ^b^	144.87 ± 7.06 ^bc^
PM153	3.73 ± 0.04 ^b^	189.83 ± 0.00 ^a^
PM177	3.81 ± 0.11 ^b^	174.84 ± 21.19 ^ab^
BCRC10696	4.09 ± 0.09 ^ab^	139.88 ± 0.00 ^bcd^
BCRC14625	4.25 ± 0.07 ^a^	104.91 ± 21.09 ^cd^
BCRC14759	4.03 ± 0.37 ^ab^	99.91 ± 28.26 ^d^
BCRC17010	3.77 ± 0.07 ^b^	199.82 ± 14.13 ^a^

^a–d^ Value in the same raw with different letters indicate significant difference (*p* < 0.05).

**Table 2:** The inhibitory effects of the MRS medium under difference pH values and l-lactic acid levels on the growth of HT-29 cell lines using MTT assay.

Inhibition Ratio (%)					
l-Lactic Acid Levels	10 mM	50 mM	100 mM	150 mM	200 mM
pH 4.5	58 ^a^	113 ^b^	155 ^c^	155 ^c^	155 ^c^
pH 5.5	60 ^a^	45 ^a^	103 ^b^	109 ^b^	149 ^c^
pH 6.5	73 ^a^	95 ^ab^	71 ^a^	97 ^ab^	116 ^c^
pH 7.5	60 ^a^	45 ^b^	81 ^c^	93 ^d^	116 ^e^

^a–e^ Value in the same column with different letters indicate significant difference (*p* < 0.05).

**Table 3:** The inhibitory effects of lactic acid bacteria cell free supernatant on the growth of HT-29 cell line for 24 h using MTT assay.

	Concentration	Inhibition Ratio (%)						IC_50_
Strains		200 μL/mL	300 μL/mL	400 μL/mL	500 μL/mL	600 μL/mL	700 μL/mL	
BCRC17010		−39.2	−6.5	45.2	73.0	75.6	99.9	479.2
BCRC10696		−81.9	−84.2	−31.7	44.1	63.5	58.5	609.8
BCRC14625		25.7	39.8	55.1	71.0	78.4	99.9	370.7
BCRC14759		4.5	24.5	43.8	62.3	63.3	43.5	467.9
PM150		−227.6	−176.9	−94.9	−42.7	23.6	52.6	667.5
PM153		27.1	50.6	75.0	86.5	89.4	100.0	299.3
PM177		56.6	62.0	74.4	84.3	88.4	99.9	134.9
